# Plants in Microgravity: Molecular and Technological Perspectives

**DOI:** 10.3390/ijms231810548

**Published:** 2022-09-11

**Authors:** Abu Imran Baba, Mohd Yaqub Mir, Riyazuddin Riyazuddin, Ágnes Cséplő, Gábor Rigó, Attila Fehér

**Affiliations:** 1 Umeå Plant Science Centre, Department of Forest Genetics and Plant Physiology, Swedish University of Agricultural Sciences, SE-901 83 Umeå, Sweden; 2 Doctoral School of Neuroscience, Semmelweis University, H-1083 Budapest, Hungary; 3 Theoretical Neuroscience and Complex Systems Group, Department of Computational Sciences, Wigner Research Centre for Physics, H-1121 Budapest, Hungary; 4 Department of Plant Biology, Faculty of Science and Informatics, University of Szeged, Közép fasor 52, H-6726 Szeged, Hungary; 5 Biological Research Centre (BRC), Institute of Plant Biology, Eötvös Loránd Research Network (ELKH), H-6726 Szeged, Hungary

**Keywords:** microgravity, Arabidopsis, amyloplast, auxin, gravity sensing, international space station

## Abstract

Plants are vital components of our ecosystem for a balanced life here on Earth, as a source of both food and oxygen for survival. Recent space exploration has extended the field of plant biology, allowing for future studies on life support farming on distant planets. This exploration will utilize life support technologies for long-term human space flights and settlements. Such longer space missions will depend on the supply of clean air, food, and proper waste management. The ubiquitous force of gravity is known to impact plant growth and development. Despite this, we still have limited knowledge about how plants can sense and adapt to microgravity in space. Thus, the ability of plants to survive in microgravity in space settings becomes an intriguing topic to be investigated in detail. The new knowledge could be applied to provide food for astronaut missions to space and could also teach us more about how plants can adapt to unique environments. Here, we briefly review and discuss the current knowledge about plant gravity-sensing mechanisms and the experimental possibilities to research microgravity-effects on plants either on the Earth or in orbit.

## 1. Introduction

Plants are an important element of life support systems in space exploration due to the fact that they provide essential components for the long-term extra-terrestrial survival of humans. They can be used in bio-regenerative life support systems (BLSS) as source of food and O_2_, for the removal of CO_2_, and for the recycling of waste during space missions. As well as improving the atmosphere of closed environments, plants can also provide good psychological health benefits for astronauts [1,2,3,4]. However, plants must be able to adapt to and grow in extra-terrestrial environments to provide the above functions.

Plants respond to environmental signals and changes by reorienting their growth. They adjust their orientation through the differential growth of their various regions [5]. Such directed growth responses in plants are termed tropisms [6]. Plant shoots generally grow against the gravity vector and towards light to support photosynthesis, whereas roots grow in the direction of gravity and away from light to acquire the water and nutrients from the soil [7]. Unlike other changing biotic and abiotic conditions, gravity is the sole stable environmental factor to which plants had to adapt during their evolution [8]. While land plants have evolved by adaptation to a gravitational force equal to 1-*g*, the growth of aquatic species is adjusted to a lower value [9]. The gravitropic signaling and response mechanism of plants grown on Earth has long been an area of interest to researchers [5,7,10,11]. How these mechanisms influence plant growth-orientation under extra-terrestrial conditions is less understood. In an experiment, the gravi- and phototropic responses of *Arabidopsis thaliana* (L.) Heynh. (Brassicales: Brassicaceae) plants are investigated under red- and blue-light irradiation at microgravity settings of 0.1 *g*, 0.3 *g*, and 1.0 *g*, modelling environmental conditions on the moon (0.17 *g*) or Mars (0.38 *g*), respectively [12]. This study shows that directional light could act as a signal to govern directional plant growth even in the case of long-term low-gravity conditions. Nevertheless, further understanding of the molecular processes underlying gravity sensing could help to predict and/or manipulate plant behaviour under microgravity conditions. Here we review the current knowledge on the root gravitropism in the model plant *A. thaliana*, investigate what is the best-studied system, and review the various experimental systems that can be used to investigate the effect of microgravity on plant behaviour.

## 2. Molecular Aspects of Plant Gravity Responses on Earth

Gravity, as a force, is the steadiest external stimulus on Earth that influences plant growth and development, and hence total plant performance. The typical reaction of plants to gravity is the growth of roots towards and the shoots against the gravity vector [13]. Differential cell elongation induced by gravity and mediated by auxin in proximal and distal tissues results in the downward curvature of roots to acquire water and nutrients and the upward curvature of shoots to reach for sunlight [14]. Primary roots/shoots usually tend to grow vertically, but lateral roots (LR) and shoot branches are oriented at a different angle to the gravity vector. This angle is denoted as the gravitropic set-point angle (GSA) that is characteristic for plant species [15]. The degree of the GSA of lateral organs is set by environmental signals and developmentally controlled gravity-sensing and response mechanisms [16].

**Figure 1:**
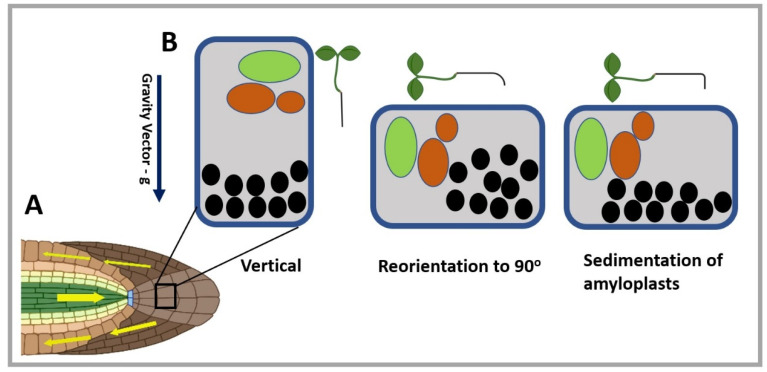
Representation of gravity-sensing according to the statolith hypothesis. (**A**) Root tip (primary site of gravity-sensing) representing the horizontal orientation of root and auxin flow. Yellow arrows show the auxin flow after stimulation by gravity. (**B**) Gravity-sensing in the columella cell (primary site of gravity-sensing or perception in the root) along the gravity vector, indicated by the blue arrow pointing downwards. On reorientation of the seedling, amyloplasts (black circles) sediment in the cell along the new gravity vector, causing the root bending. The green region represents the nucleus of the columella cell and the orange region the vacuoles. (Some segments of the figure were created with BioRender.com) (accessed on 2 June 2022).

The molecular mechanism of plant gravity-sensing and response has been an important topic of debate in past decades raising several hypotheses. Gravity-sensing in plants was first explained by the still widely accepted starch (amyloplast)–statolith hypothesis [17,18]. Statoliths are membrane-bound starch grains (amyloplasts) in gravity-detecting cells that can be found in the shoot cortex and the root columella [19]. The basis of the theory is that statoliths sink to the bottom of reoriented gravity-sensing cells due to the change in the gravity vector (Figure 1). According to the hypothesis, the settling of amyloplasts is the primary component of gravity-sensing that triggers downstream signaling, generating biochemical and physiological responses in the responsive plant tissues. A less popular gravitational pressure model states that the whole protoplast (the entire cell content) has a role in sensing gravity [20]. This model explains that mutants lacking starch in their plastids still respond to gravity, although with a reduced sensitivity [21]. However, the importance of statoliths in gravity-sensing could not be questioned, as it was supported by several other experimental observations [17]. A recent hypothesis, the graviproprioceptive drive (GPD) theory, explains the limited but still important role of statoliths [22]. Some models indicate that statolith movement and cell reorientation are in continuous feedback, resulting in oscillating growth. Based on this, the GPD theory proposes that the straightness of the stem is attained by gravicline-sensing provided by statoliths together with the proprioception of tissue bending (“self-sensing of tension and deformation”) [22].

The mechanism of organ bending in response to directional signals (tropism) was proposed by Cholodny (1927) [23] and Went (1926) [24], (referred to as the Cholodny–Went hypothesis). According to their model, the gravitropic response, for example, relies on the lateral transport of auxin establishing unequal concentrations at the two sides of the organ (towards and away from the gravity signal), resulting in differential cell elongation and organ bending [5,14,25].

The role of auxin transport in gravitropism was underlined by applying the auxin transport inhibitor, NPA (naphthylphthalamic acid), preventing gravitropic root bending [26]. Secondary metabolites such as flavonoids act as endogenous negative regulators of auxin transport. The mutation *transparent testa* (*tt4*) that disrupts a gene that encodes for chalcone synthase (CHS), the first enzyme of flavonoid biosynthesis, leads to an increase in root growth and gravitropism due to increment of auxin transport [27]. The *tt4* mutants have substantially higher levels of transcripts of the PIN-FORMED (PIN) protein 2 (PIN2) than the wild type, which aids in basipetal auxin transport in the root [28]. Auxin and ethylene hormone interaction controls flavonol production via a signalling network involving TRANSPORT INHIBITOR RESPONSE1 (TIR1) and ETHYLENE INSENSITIVE2 (EIN2)/ETHYLENE RESISTANT1 (ETR1), which both converge on the MYB12 transcription factor [29].

The gravity-induced asymmetrical auxin distribution at the root tips is established and maintained by various auxin influx and efflux transporters, including the AUX1/LAX (AUXIN-RESISTANT MUTATION 1/LIKE AUX1) proteins, the ATP BINDING CASSETTE B/MULTIDRUG-RESISTANCE/P-GLYCOPROTEINS (ABCB/MDR/PGP), and the PIN-FORMED (PIN) proteins [30,31]. The polar distribution of auxin is primarily governed by the PIN auxin efflux carriers, which are polarly localized in the cell membrane, pumping the auxin only in a defined direction [32]. The PIN1,2,3,4, and -7 proteins collectively control auxin distribution in the primary root [33]. These proteins are present in specific, but overlapping, root tip regions [34]. The PIN1 mainly resides in the basal membrane of vascular parenchyma cells, and contributes to the accumulation of shoot-derived auxin in the root meristem. An incorrect expression or localization of PIN1 in *hsp90* mutant roots prevented the gravitropic response [35]. The PIN2 is localized apically in the PM membranes of epidermal and lateral root cap (LRC) cells and basally in cortical cells. Thus, PIN2 transports auxin towards the shoot in epidermal cells but towards the tip in cortical cells, generating an auxin transport loop that was found to be important for the gravitropic response [34]. The PIN2 mutants (designated as *eir1*/*agr1*/*pin2*/*wav6*) exhibited an impaired root gravity response [13,26]. The PIN3 and PIN7 are expressed in the LRC columella cells without pronounced polarity, and are also present in specific regions of the meristem. FOUR LIPS (FLP) controls PIN3 and PIN7 transcription levels in *A. thaliana* [36]. Consequently, the auxin level was lower in the gravity-sensing cells of the *flp* mutant compared with wild-type roots [36]. The PIN4 can be observed in cells surrounding the quiescent centre and in the basal membrane of provascular cells. While *pin2* mutants exhibited an agravitropic phenotype, the *pin3*, *pin4,* and *pin7* mutants were strongly gravitropic. Nevertheless, all these members of the PIN family have been related to the root response to gravity, probably with partly redundant roles [31]. According to the current model, upon change in the orientation of the root, the PIN3 and PIN7 transporters relocalize towards the lower side of the plasma membrane, directing auxin flow into the lower tiers of LRC cells. Once asymmetry in auxin accumulation is initiated by PIN3 and PIN7, the PIN2 transporter asymmetrically accumulates in the upper and lower sides of the root [37,38]. As of consequence, auxin is transported in higher quantities in the lower epidermal cell tiers by PIN2, thus inhibiting cell elongation that results in the downward turning of the growing root [39].

The polar localization of PIN proteins at specific membrane domains is a dynamic process due to their endocytic vesicular recycling [40,41,42]. The PIN polarity is controlled by posttranslational modifications (phosphorylation and ubiquitination) [40]. PINs are phosphorylated at specific residues by specific kinases [43]. These kinases include the PINIOD (PID), the WAG2 [43,44], and the CDPK-RELATED KINASE5 (CRK5) [37,45,46,47,48]. Both loss-of-function and gain-of-function mutants of PID exhibit defective PIN3 polarization, and thus prevents bending in response to gravity [49]. The PIN3 phosphorylation is, therefore, essential for the gravitropic response [50]. Furthermore, the PIN2-dependent basipetal auxin transport is also reduced in *pid-9* mutant roots [51]. The CRK5 was shown to be able to phosphorylate the hydrophilic loop of PIN2, and the At*crk5* mutant also exhibited a delayed root gravitropic response, correlated with inhibited redistribution of PIN2 and limited auxin accumulation in the root tip region [37]. The CRK5 kinase can also phosphorylate the hydrophilic loop of other PIN proteins, including PIN3 [46]. Recently, AtCRK5 was shown to have a role in maintaining the balance between reactive oxygen species (ROS) and nitric oxide (NO) during root gravitropism in *A. thaliana* [38]. The asymmetric redistribution of both ROS- and NO-production is induced by auxin during the gravitropic response of roots [52,53], and both were shown to affect PIN2 turnover and consequently auxin transport [54,55]. Based on these observations, a regulatory feedback loop involving auxin, ROS, and NO operating during the early gravitropic response of roots was hypothesized [38].

**Figure 2:**
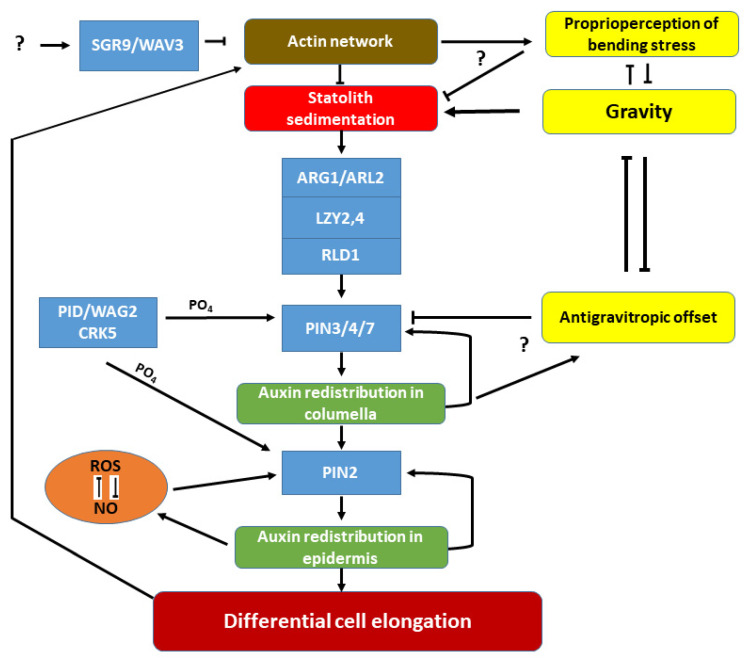
Simplified hypothetical model depicting some of the known components of gravity-signalling and -response pathways in plants, mostly based on data obtained on *A. thaliana*. The organisation of the actin network controls statolith sedimentation in the reorientating of the gravity-sensing cells. Protein degradation via the SGR9/WAV3 and related ubiquitin ligases hampers this control. Sedimenting statoliths initiate cellular polarization via the ARG1/ARL2-LZY2,4-RLD1 membrane-associated protein module. This module relocalizes the PIN3/4/7 proteins to the membrane domain towards the gravity vector (only PIN3 is expressed in the emerging lateral root, but all three in the columella of the main root). Auxin is redistributed in the gravity-sensing cells, and the asymmetric basipetal transport of auxin at the lower side stabilises PIN2 in the basal membrane, pumping auxin towards the shoot in higher quantities in the lower epidermis (towards the gravity vector). This prevents cell elongation in these cell rows, resulting in differential cell elongation between the two sides of the root causing downward bending. The polar localization of PIN proteins is dependent on their phosphorylation by various protein kinases (PID/WAG2/CRK5). Asymmetric auxin distribution establishes asymmetry in ROS-NO accumulation. ROS and NO are in an intricate relationship, and their balance is required to maintain PIN2 stability in the membrane. The effect of gravity is counterbalanced by the hypothetical proprioperception of mechanical stress caused by organ bending (likely involving the actin network) and by the auxin transport-dependent antigravitropic offset (molecular components are yet unknown) contributing to the gravitropic set-point angle of lateral organs.

The role of cytoskeleton in plant gravitropism attracts attention from time to time [56]. According to the tensegrity (“tension and integrity”) model of gravitropism, the plant cytoskeleton acts as a possible sensor and transmitter of the gravitropic signal [56]. Microtubules, filamentous actin (F-actin), and numerous regulatory proteins make up the cytoskeleton system. Using microtubule or actin polymerisation inhibitors in plants has highlighted the importance of the cytoskeleton in gravitropism [55,57,58,59,60,61,62,63,64]. However, the significance of actin in gravitropism is debated, since disruption of the actin network did not always result in an agravitropic phenotype but could even enhance statolith sedimentation and the gravitropic response [56,65]. The actin cytoskeleton is therefore believed to not be crucial for gravity-sensing per se, but for controlling the resting and sedimentation of statoliths [56]. In agreement, the plastid-localized SHOOT GRAVITROPISM RESPONSE 9 (SGR9) C3H2C3 ring finger protein with ubiquitin E3 ligase activity was shown to be required for amyloplast dissociation from the actin filaments [66]. The *sgr9* mutant exhibits reduced gravitropism since its amyloplasts cannot sediment. In contrast, in the *fiz1* Arabidopsis line having fragmented actin filaments due to a mutant ACTIN8 gene, the statoliths settle almost without hindrance [66]. The ubiquitin ligase activity of SGR9 was found to be necessary for gravity-sensing, involving the protein degradation mechanism in the actin-mediated regulation of statolith dynamics (Figure 2). This view is strengthened by the fact that another E3 ubiquitin ligase, WAVY GROWTH 3 (WAV3), was also shown to be required for the root gravitropic response [67]. The homologs of WAV3, including EDA40, WAVH1, and WAVH2, have redundant positive functions in gravitropism, and the *wav3 wavh1 wavh2* triple mutant revealed auxin-signalling defects during the gravitropic response in roots [67]. The identification of the protein targets of E3 ligases implicated in gravitropism could highlight important details linking statolith dynamics to auxin distribution. Recent discoveries led to the hypothesis that members of the LAZY1-LIKE (LZY) family of proteins have an important role in this link [50]. The phenotypic characterization of four members of the *A. thaliana LAZY* gene family revealed that the four members expressed in statocytes in both the shoot and the root (*LZY1–3* in shoot and *LZY2–4* in root statocytes) contribute to gravitropism, as well as to the setting of the GSA [50,68,69]. The defect in the gravitropic bending of *lzy* multiple mutants was due to their impairment in lateral auxin redistribution without the impact on amyloplast sedimentation on realignment [69].

*A. thaliana* lateral roots emerge perpendicular to the primary root, but soon attain a GSA of approximately 75° [70]. This is due to the interaction of gravitropic and antigravitropic offset (AGO) growth components, which are both auxin transport-dependent [71,72]. The results suggest that GSAs are maintained by the interaction of the two opposing auxin fluxes, the balance of which determines the angle of organ growth. In agreement, following their emergence, lateral roots maintain a gravity-dependent non-vertical growth that progressively turns to vertical over several days. During this growth period, the lateral roots can reorientate their growth either upwards or downwards to maintain their GSA. In the lateral root tips of *lzy* mutants, the asymmetric distribution of PIN3 and auxin response were reversed, suggesting that LZYs control asymmetric PIN3 accumulation and the direction of polar auxin transport (PAT) in the root cap columella in response to gravity [68]. An analysis of the GSAs of the lateral roots of *pin3-4*, *pin4-3*, and *pin7-2* single and multiple mutants led to the hypothesis that the repression of PIN4 and PIN7 in the gravity-sensing columella cells is the cause of the limited gravitropic response of young lateral roots, in comparison with the main root where all three PINs (PIN3/4/7) are expressed in the statocytes [73]. This is believed to strengthen the gravitropic response, allowing for the predominantly vertical growth of the main root. Recently, the LZY-interactor RCC1-like domain (RLD) proteins were also reported as essential regulators of PAT and GSA [74]. The following model emerged: statolith sedimentation first results in the polarization of the cellular localization of LZY that recruits RLD1, which in turn is required for the relocalization of PIN3 modulating the auxin flow [69,74]. The transmembrane protein, ALTERED RESPONSE TO GRAVITY 1 (ARG1), and its paralog ARG1-LIKE2 (ARL2) were also implicated in PIN3 relocalization and asymmetrical auxin distribution during gravistimulation [7,75,76]. Both *arg1* and *arl2* mutations exhibit slower reorientation of hypocotyls and roots, without affecting the root starch content [76,77]. It was hypothesized that these membrane-associated DnaJ proteins might have a role in the cellular polarization of LZY [69]. The current molecular model of sensing gravity in roots is schematically summarized in Figure 2.

## 3. Plants in Simulated or Real Microgravity Conditions

### 3.1. Experimental Platforms

**Figure 3:**
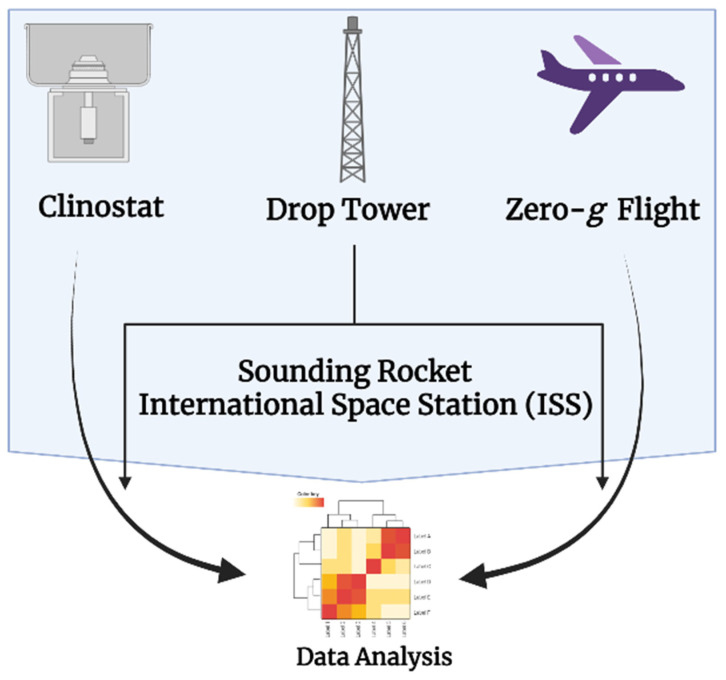
Pictographic representation of some platforms used for microgravity simulations on Earth and lower orbit. Arrows indicate the samples from different platforms that are collected and sent for experimentation and data analysis (Figure was created with help of BioRender.com) (accessed on 2 June 2022).

Plants will be crucial in ensuring resources for human existence on long-duration journeys beyond Earth during space colonization. Microgravity conditions in space offer an unparalleled environment to study reduced gravity’s effects on animal and plant organisms. Plants in space may not only supply future food, but also oxygen for survival. Plant experiments have been performed in space since the launch of Soviet/Russian Salyut 1, the first space station [78]. Plants have also been cultivated in various space-mimicking environments for a long time now [78]. Therefore, the research on plant growth in space conditions has a detailed history. The long-term aim is to develop a bioregenerative life support system that can provide astronauts with fresh food, oxygen, decreased CO_2_, and recycled metabolic waste [79,80,81]. As a food source in bioregenerative life support systems, plants may be affected by the long-term impacts of the space climate [82]. To cultivate plants successfully in space, we must first deeply understand plant growth mechanisms and behaviour in microgravity situations [83]. Gravity regulates buoyancy, convection, and sedimentation, which all influence a range of physical and chemical processes also associated with plant growth and development [84]. The ubiquitous force of gravity impacts plant growth, development, and morphology at all levels [85]. To study this, plants have been subjected to microgravity or hypergravity environments in labs on Earth and the International Space Station (ISS) platform [80]. Here on Earth, short-duration microgravity conditions are simulated by approaches such as rotation in clinostat, free fall in drop tower experiments, parabolic flights using airplanes, and the sounding rockets, as pictographically represented in Figure 3. Long-term microgravity, however, can only be perceived by plants in experiments performed in orbit on ISS. These reported experimental platforms have indeed a different quality and duration of microgravity stimulations. However, access to some of these experimental platforms, including the ISS, is still limited and very costly. Here, we briefly describe some of these methods used to research plant gravity perception and experimentation, mostly under fractional or long-term microgravity conditions.

#### 3.1.1. Clinostats

A clinostat, developed in the 19th century, is a device using rotation to study the effects of a nullified gravitational pull on plant growth and development [86]. Clinostats are named based on rotational axes: one-axis clinostats with slow (1–4 rpm) or fast (50–120 rpm) rotations, and clinostats with multiple axes of rotation. Modified clinostats with changing rotation axes are defined as random positioning machines (RPMs) [87]. To study the impact on plants, seeds, or small plantlets, those are grown or attached to solid or semi-solid based media and placed into these rotating devices [88]. Clinostats can be used to eliminate the effect of constant gravity on objects imitating brief periods of microgravity [89]. Since scientists discovered gravity as a key factor to plant growth and development in the late 1800s, clinostats have been created and used for such studies [90]. Several of these studies have shown the utility of comparative analysis based on the biological impacts of clinostats and specific microgravity methods for space research [91,92,93]. Most artificial gravity reactions during shorter time periods can be explored using clinostats and validated further by using free-fall studies [94]. During clinostat rotation, the force of gravity does not change over time, but differential growth will cause plant organs to bend and shift the organ position in long-run experiments. This must be taken into consideration when comparing the results obtained with different axes [88,95]

#### 3.1.2. Drop Towers

Drop towers are another form of ground-based facility used for experiments to study the impact of microgravity on plants, as well as other scientific experiments. This approach mostly produces a few seconds of weightlessness on objects [96]. It is a low-cost method on the ground to mimic short durations of microgravity on plants, and involves an experimental payload catapulted through the vacuum to reduce air friction [97]. Short microgravity exposure in a drop tower is obtained when a catapult-driven container with a biological sample falls freely. Drop tower samples experience microgravity to the level of 10^−6^ *g*, and allow for the precise monitoring of experimental data during this course. Drop tower, for instance the one based in Bremen, Germany, consists of a cylinder-shaped capsule with a specific diameter, height, and mass dropped at approximately 110 m vertically on the ground [97]. Actually, the duration of microgravity on this platform is limited, and cannot provide data on the effect of long-term diminished gravity perceived by plants and their adaptation to such environments.

#### 3.1.3. Parabolic Flights and Sounding Rockets

Parabolic flights use special airplanes that generate approximately 10–20 s of microgravity on carrying objects. Such aviation-based parabolic flights use an airplane designated to perform 31 parabolas in microgravity every 22 s [96]. Parabolic flights can also be used to investigate the effects of alternating microgravity and hypergravity phases on gravireceptor activation in specific plant cells [98]. It must be taken into consideration that the samples on parabolic flights are exposed to both the hyper- and microgravity conditions after the first parabola. Thus, the samples collected from such experiments have experienced both conditions at different time points of flight, and this might affect the result obtained [96].

Another instrumentation to generate a longer duration of microgravity on objects includes the sounding rockets. They may provide another option for short-duration microgravity simulation, producing minutes of weightlessness on samples. During the free-fall, these trips on rockets provide 4–13 min of overall microgravity conditions [96]. These rocket programs have also contributed to several experimental programs involving physical and biological sciences. However, the weightlessness time is again too short to effectively assess long-term growth and development, as in most of these discussed short-duration simulators.

#### 3.1.4. Space Flights and the International Space Station

The difficulty of altering gravity on Earth complicates research into gravity’s direct and indirect effects on plant development. The microgravity research station on orbit is known as the “International Space Station” (ISS), which provides a place to perform experiments related to microgravity. The ISS includes a low-orbit space station for long-term gravity experiments, and is equipped with laboratories for research and investigation in various scientific fields. The ISS does not have artificial gravity services; therefore, the European Modular Cultivation System (EMCS) on board on the ISS conduct experiments on plants and animals. Moreover, to perform the plant-signalling flight experiments, the ISS has the EMCS, which contains two rotors within a controlled chamber that permits for experimental conditions such as microgravity and simulated gravity in space. Before spaceflight platforms, it was impossible to investigate the long-term physiological changes caused by low gravity [80]. On the ISS platform, crew members work in laboratories conducting scientific research on samples. The inclusion of plant-growing facilities on orbiting space platforms such as the ISS allows plant researchers to conduct complex long-term microgravity studies in space. Less than a decade ago the first plant growth chamber called ¨Veggie¨ was built to grow and experiment with plants in space on the ISS. [80]. Parts of investigations have shown how plants react to spaceflight circumstances, including the technological and operational advancements necessary to perform reliable biological research in space [99]. In all instances, the space-derived experimental results on the ISS platform must be validated with control samples and experiments on Earth. Previous research on microgravity simulators/analogs raised some concerns about matching experimental results obtained in space to simulation results [92]. Therefore, the conditions allowing ground-based simulations to compare with gravitational regimes generated on the ISS must be carefully specified through well-controlled experiments. For example, both space and ground experimental containers and growth cabinets should be equally controlled in climatic, light, culture media constitution, watering regime, and other environmental conditions. One of the other limitations is that space agencies are reluctant to share their expensive spaceflight hardware because of the limited available space [100].

Microgravity-based plant growth system’s main concerns are irrigation to maintain moisture at the root level while avoid flooding and anoxia and the selection of lighting systems and suitable plant species for space farming [101]. There have already been several reports on attempts to cultivate crops in space stations. As first attempts, leeks, onions, and Chinese cabbages were planted on Salyut 1, a Soviet Union space station, in the year 1971 (Oasis production system) [102]. The cosmonauts Klimuk and Sevastianov ate the first space-grown vegetables, onions, in space in 1975 [102]. In recent years, red romaine lettuce, *Lactuca sativa* L. cv. Outredgeous (Asterales: Asteraceae) plants were grown in growth chambers (Veggie production system) to study the impact of microgravity on microbiological and nutritional quality of vegetables grown in space [103]. These veggies have also been involved in space crew’s supplementary diets [103]. Even more recently, American astronauts were eating chili peppers grown and harvested on the ISS (https://www.businessinsider.com/nasa-world-records-chili-peppers-international-space-station-2021-12; accessed on 12 June 2022). In summary, the long-term experimental approaches such as those performed on the ISS will provide deeper details about molecular adaptations and responses of plants to microgravity. However, some conclusions can also be drawn from the experiments performed in shorter durations of microgravity.

## 4. Advancements in Sample and Data Analysis

The collected materials from different ground-based instruments and space platforms are sent for further laboratory examinations. Previously, experiments aimed to determine the morphological and physiological consequences of microgravity on plants. Currently, samples are mostly collected from various experiments for transcriptomic, proteomic, phosphoproteomic, and metabolomic studies [104,105,106,107,108,109]. For this purpose, specific tools and protocols have been elaborated and designed. For example, the samples for RNA collection utilizes the KSC fixation tubes to collect and preserve samples from different platforms [110] and utilizes RNA-Later, a tissue storage reagent, used as fixative to stabilize and protect RNA during the delivery of samples and allowing for them to be stored until further investigations [111]. Furthermore, the increasing number of such experimental data and results has also led to the creation of GeneLab (https://genelab.nasa.gov/) (accessed on 12 June 2022), a repository for molecular “omics” data from spaceflight and corresponding analogue experiments. Most importantly, the use of novel single-cell experimental systems has also provided an easier and more advantageous platform to experiment with microgravity responses at the single-cell level. The observations and the handling of cells on the microscopy platform at the single-cell level are easier compared to the whole plant in altered gravity conditions.

## 5. The Reaction of Plants to Microgravity

The response of plants to microgravity has already been the subject of many investigations, since reduced gravity can impact the water and nutrient uptake of plants, affecting their growth and yield [101]. Recent research has resulted in new insights into plants’ physiological, biochemical, molecular, and growth characteristics under space flight conditions [81]. E.g., studies with the Micro-Tom dwarf tomato variety highlighted the negative effect of microgravity on nutrient acquisition and plant growth, including the overall yield [112]. The influence of microgravity on plant performance is dependent on the exposure duration: short-term responses are characterized by the activation of various signalling pathways, including Ca^2+^, lipid-, pH-, reactive oxygen species (ROS), and auxin-signalling and changes in the metabolic profile [113]. Experiments using root explants and plantlets from tissue cultures have also been previously carried out in space, but no molecular investigations were performed [114,115]. The fern single-cell model system of spores of *Ceratopteris richardii* Brongn. (Polypodiales: Pteridaceae) appeared to be particularly susceptible to space flight at the molecular level, showing responsiveness to the gravity vector [116]. The samples analysed for phytohormone detection have revealed changes in the contents of jasmonate, auxin, and numerous cytokinins during hypergravity and microgravity reactions [117]. Experiments with seedlings of the cucumber, *Cucumis sativus* L. cv. Burpee Hybrid II (Cucurbitales: Cucurbitaceae) in microgravity showed that PIN1 relocalized on gravistimulation, redirecting auxin to the lower side in endodermal cells [118,119]. In another experiment, the growth of *Zea mays* L., 1753, cv. Golden Cross Bantam (Poales: Poaceae) in microgravity situations in space improved the polar transport of auxin. These changes enhanced PAT in maize shoots are the consequence of the changes that occurred in Zm*PIN1a* levels and its polar localization in the coleoptiles [120]. Changes have also been observed in the plant cell cycle [121] and cell shape [122] under microgravity.

Detailed spaceflight experiments have also examined the impact of varying gravity levels on gene expression in plants [123,124,125]. The adaptation of plants to space flight conditions involves the induction of some known and unknown stress-related genes [124,126,127]. The comparative spaceflight experiment for transcriptome analysis in the root tips of the *Phytochrome D* (*PhyD*) mutant of *A. thaliana* exhibited a significant decline in transcriptome response in comparison with wild-type controls [124]. The analysis of microarray data indicated that the PIN3-dependent regulation of auxin-responsive genes in temporary microgravity is mediated by PIN2 [128]. During microgravity, the expression pattern of genes associated with oxidative stress and cell wall remodelling was also found to be altered [107]. An investigation of the first pieces of evidence for alternative splicing during spaceflight involved the RNA sequencing of the samples from *A. thaliana* during the APEX03 experiment [129]. The results from this experiment displayed significant differences in alternatively spliced isoforms in comparison with the ground controls. Specific green-fluorescent reporter (GFP) gene expression analysis using confocal microscopy for auxin and cytokinin distribution on the ISS platform displayed a vertical arrangement of auxin hormone in the primary root [130]. Furthermore, the analysis of the gene expression reporter of the other hormone, the cytokinin, during such conditions in root tip varied in samples grown on ISS compared with specific ground controls [131]. Another study identified different transcription factors that displayed a pattern of increased transcript profiles on simulated gravity platforms [131]. A study reported the effect of fractional gravity on the 5-day-old seedlings, and observed changes in the transcripts of genes that were related to defence, cell-wall associated genes, and heat shock genes (*HSP70*, *HSP90.1*, *HSP101*, and the DNA J domain) [132]. The change in transcripts was mostly influenced by the exposure of seedlings from 0.53 *g* to 0.88 *g* compared with control conditions [132].

Other than the long-term effects of the ISS platform, partial or reduced gravity experiments have been included to test the diminished effects of gravity [131,133]. An experiment involved the *A. thaliana* seeds germinating and grown under simulated gravity similar to that of the moon’s (0.17 *g*) and Mars’ (0.38 *g*) gravity. The results of these partial gravity simulation experiments displayed an increment in cell proliferation and decreased cellular growth under the levels of moon gravity [133]. In such simulated microgravity conditions, the *A. thaliana* cell culture showed an increased speed in the cell cycle due to a shorter G2/M phase and little increment in the G1 phase [134]. This also led to changes in the necessary nuclear function of the cells.

Moreover, microgravity influenced photosynthesis process in various plants species, as reported in *Brassica rapa* (Capparales: Brassicaceae) [135], rice seedling *Oryza sativa* L. (Poales: Poaceae) [136], *Ipomoea batatas* (L.) (Solanales: Convolvulaceae) [137], and *Hordeum vulgate* L. 1753 (Poales: Poaceae) [137]. For example, Kitaya and co-workers reported higher photosynthesis rates in *I. batatas* (L.) [137] and *H. vulgare* plants species with increasing gravity from 1.0 *g* to 2.0 *g*, but a reduced photosynthesis rate was reported with decreasing gravity from 1.0 *g* to 0.1 *g* [137]. In the case of rice seedlings, chlorophyll content was improved in the exposure of rice plants to clinostat at 2 rpm for up to 7 days, while chlorophyll content was decreased after exposure to the rice plants for more than 7 days [136]. A recent study reported the seed germination of *Pinus pinea* L., 1753 (Pinales: Pinaceae) under altered gravity, and found a higher seed germination rate under microgravity (2 × 10−3 *g*) than in hypergravity (20 *g*) conditions [138]. This was due to a higher activity of enzymes related to seed germination, such as 3-HADH (3-hydroxyacyl-CoA dehydrogenase) (fatty acids oxidation), ICL (isocitrate lyase), MS (malate synthase) (glyoxylate cycle), isocitrate dehydrogenase (ICDH; Krebs cycle), glucose 6 phosphate dehydrogenase (G6PDH; pentose phosphates shunt), and pyruvate kinase (PK; glycolysis) [138]. Similarly, the exposure of *P. pinea* seed to hypergravity for 64 h at 4 °C hampered the enzyme activity associated with seed germination and subsequently delayed the germination of seeds of the same species as compared with control conditions [139]. Microgravity had a negative impact on seed viability and germination of *Eruca sativa* Mill. (Capparales: Brassicaceae) that were stored on the ISS for 6 months compared with those held on the Earth’s surface [140]. Likewise, lower seed germination, decreased plant height, a smaller number of tillers, and an altered expression of associated protein D14 were reported in rice seeds under orbited SJ-10 spaceflight for 12.5 days [141]. A recent study by Villacampa et al. (2021), reported that the three gravitropism stages, statolith sedimentation, asymmetrical auxin distribution, and differential elongation, that regulate *A. thaliana* seedling growth were differentially modulated under fast and slow horizontal and vertical clinorotation conditions [142]. Moreover, the flavonoid synthesis pathway mutants known as *transparent testa* (*tt*) grown in the presence of light or a D-ROOT device showed stunted growth of shoots and roots as compared with the wild type. However, in the *tt3* mutant, flavonoid-accumulation plants exhibited better growth and proper development of the longitudinal axis when grown in a D-ROOT device under dark growth conditions as compared with wild-type *tt4* (flavonoid-deficient) mutants [143]. This concluded that the proper re-establishment of root directional growth to achieve proper water and nutrients in *A. thaliana* plants under simulated microgravity depends on the flavonols that regulate the light-avoidance mechanism [143]. A relatively new device equipped with an LED lighting system designed for plant experiments in the large diameter centrifuge (LDC) gondolas and the large-size random positioning machine (RPM) (ROOTROPS) was used to investigate the impact of simulated gravity, along with light quality, on the root orientation of crop of the plant *Brassica oleracea* var. italica (Brassicales: Brassicaceae) seedlings. The vigorous growth of the *B. oleracea* seedling was reported in combination with different light regimes (red, blue, and white) and simulated RPM to centrifugation up to 20× *g* under the dark-grown conditions [144]. Similarly, the growth rate of *Abelmoschus esculentus* (L.) Moench (Malvales: Malvaceae) seeds was increased up to 14% when grown under simulated microgravity rather than in the Earth’s gravity [145]. Moreover, simulated microgravity reduced the expression of the *PMEPCRA* gene in *A. thaliana* responsible for the biosynthesis of the pectin methylesterase enzyme involved in pectin remodelling and cell wall decomposition that mechanically helps plants to acclimatize during simulated and spaceflight microgravity [146]. While altered phenotypic growth parameters were observed in the *Atpmepcra* mutant plants compared with the wild type, this might be due to the alteration of pectin stiffening. Similarly, simulated microgravity altered the composition of cell walls, such as reducing the ratio of polysaccharides and protein in the *Nicotiana tabacum* cv. Burley 21 (Solanales: Solanaceae) plants, enhancing the potential of cells to perform cell division and expansion at a higher rate [147]. Hence, we can conclude from such experimental inputs here that the effects of altered gravity on plant performance vary from species to species and according to the type of simulated gravity and to the time of exposure.

## 6. Concluding Remarks and Future Prospects

Gravity stimulation is a vital component for the growth and development of plants on Earth. According to the most popular theory, the overall process of gravitropism in plants involves an initial act of gravity on statocyst sedimentation followed by auxin relocalization to eventuate differential changes on cell growth [17,18]. However, there are still many details that are not known concerning the effects of gravity stimulation on the mechanistic growth and development of plants on Earth. We know, however, even less about how plants adapt in space to the challenge of microgravity environments. What is the impact of this environment on the gravity-adapted molecular control of plant growth and development? Questions about plant responses to microgravity are largely unanswered. Various experimental and technical platforms have been proposed to study plant adaptations to microgravity. The use of these platforms has added some missing pieces to the puzzle. These include highlighting some molecular signaling pathways that will further help to decipher the processes driving plant microgravity responses. Different molecular genetics approaches will inform us about the various functional and structural components of gravity signalling on Earth as well as in space. Among others, it would be interesting to understand the significance of kinase-dependent and independent mechanisms modulating PIN protein polarity under space conditions. With the advent of the use of single-cell experimental models, we can acquire more cellular-level understandings of the effects of microgravity. As gravity provides a directional cue for the bending and anchorage of roots deeper in the soil to uptake water and nutrients, studying the molecular mechanisms in microgravity can also help to study and improve the agricultural yield in harsh conditions, e.g., drought stress on the earth. The knowledge attained can also be utilized to improve plant cultivation strategies and technologies, including vertical farming on Earth. Furthermore, studying responses to microgravity can help us to gain insights into gravity perception and signal transduction that relates to the impact on the overall plant growth and development. The utilization of the novel genetic engineering tools can be implied to generate plant varieties that can withstand the space stress environments and can be involved in experiments to study plant adaptation in microgravity. Furthermore, the knowledge of the impact of microgravity on plants can be employed in the future to enable astronauts to grow their own food during long-term space missions.

## Data Availability

Not applicable.

## References

[1] Ferl R., Wheeler R., Levine H.G., Paul A.-L. (2002). Plants in space. Curr. Opin. Plant Biol..

[2] Fu Y., Li L., Xie B., Dong C., Wang M., Jia B., Shao L., Dong Y., Deng S., Liu H. (2016). How to establish a bioregenerativelLife support system for long-term crewed missions to the moon or mars. Astrobiology.

[3] Medina F.J., Manzano A., Villacampa A., Ciska M., Herranz R. (2021). Understanding reduced gravity effects on early plant development before attempting life-support farming in the moon and mars. Front. Astron. Space Sci..

[4] Wheeler R.M. (2010). Plants for human life support in space: From Myers to Mars. Gravit. Space Biol..

[5] Evans M.L. (1991). Gravitropism: Interaction of sensitivity modulation and effector redistribution. Plant Physiol..

[6] Darwin C. (1880). The Power of Movement in Plants.

[7] Su S.-H., Gibbs N.M., Jancewicz A.L., Masson P.H. (2017). Molecular mechanisms of root gravitropism. Curr. Biol..

[8] Chebli Y., Geitmann A. (2011). Gravity Research on Plants: Use of single-cell experimental models. Front. Plant Sci..

[9] Kiss J.Z., Wolverton C., Wyatt S.E., Hasenstein K.H., van Loon J.J.W.A. (2019). Comparison of microgravity analogs to spaceflight in studies of plant growth and development. Front. Plant Sci..

[10] Simmons C., Migliaccio F., Masson P., Caspar T., Soll D. (1995). A novel root gravitropism mutant of Arabidopsis thaliana exhibiting altered auxin Physiology. Physiol. Plant.

[11] Sato E.M., Hijazi H., Bennett M.J., Vissenberg K., Swarup R. (2015). New insights into root gravitropic signalling. J. Exp. Bot..

[12] Kiss J.Z., Millar K.D.L., Edelmann R.E. (2012). Phototropism of Arabidopsis thaliana in microgravity and fractional gravity on the International Space Station. Planta.

[13] Chen R., Rosen E., Masson P.H. (1999). Gravitropism in higher plants. Plant Physiol..

[14] Estelle M. (1996). Plant Tropisms: The Ins and outs of auxin. Curr. Biol..

[15] Digby J., Firn R.D. (1995). The gravitropic set-point angle (GSA): The identification of an important developmentally controlled variable governing plant architecture. Plant Cell Environ..

[16] Roychoudhry S., Kieffer M., Del Bianco M., Liao C.Y., Weijers D., Kepinski S. (2017). The developmental and environmental regulation of gravitropic setpoint angle in Arabidopsis and bean. Sci. Rep..

[17] Sack F.D. (1991). Plant gravity sensing. Intern. Rev. Cytol..

[18] Sack F.D. (1997). Plastids and gravitropic sensing. Planta.

[19] Kiss J.Z. (2000). Mechanisms of the early phases of plant gravitropism. Crit. Rev. Plant Sci..

[20] Wayne R., Staves M.P. (1996). A down to earth model of gravisensing or Newton’s law of gravitation from the apple’s perspective. Physiol. Plant.

[21] Casper T., Pickard B.G. (1989). Gravitropism in a starchless mutant of Arabidopsis: Implications for the starch-statolith theory of gravity sensing. Planta.

[22] Moulia B., Douady S., Hamant O. (2021). Fluctuations shape plants through proprioception. Science.

[23] Cholodny N. (1927). Wuchshormone und tropismen bei den pflanzen. Biol. Zent..

[24] Went F. (1926). On growth-accelerating susbtances in the coleoptile of Avena sativa. Proc. K. Ned. Acad. Wet..

[25] Young L.M., Evans M.L., Hertel R. (1990). Correlations between gravitropic curvature and auxin movement across gravistimulated roots of Zea Mays. Plant Physiol..

[26] Rashotte A.M., Brady S.R., Reed R.C., Ante S.J., Muday G.K. (2000). Basipetal auxin transport is required for gravitropism in roots of Arabidopsis. Plant Physiol..

[27] Brown D.E., Rashotte A.M., Murphy A.S., Normanly J., Tague B.W., Peer W.A., Taiz L., Muday G.K. (2001). Flavonoids act as negative regulators of auxin transport in vivo in Arabidopsis. Plant Physiol..

[28] Buer C.S., Muday G.K. (2004). The Transparent testa4 mutation prevents flavonoid synthesis and alters auxin transport and the response of Arabidopsis roots to gravity and light. Plant Cell.

[29] Lewis D.R., Ramirez M.V., Miller N.D., Vallabhaneni P., Ray W.K., Helm R.F., Winkel B.S.J., Muday G.K. (2011). Auxin and ethylene induce flavonol accumulation through distinct transcriptional networks. Plant Physiol..

[30] Blakeslee J.J., Peer W.A., Murphy A.S. (2005). Auxin transport. Curr. Opin. Plant Biol..

[31] Konstantinova N., Korbei B., Luschnig C. (2021). Auxin and root gravitropism: Addressing basic cellular processes by exploiting a defined growth response. Int. J. Mol. Sci..

[32] Adamowski M., Friml J. (2015). PIN-dependent auxin transport: Action, regulation, and evolution. Plant Cell.

[33] Michniewicz M., Brewer P.B., Friml J.Í. (2007). Polar auxin transport and asymmetric auxin distribution. Arab. Book.

[34] Blilou I., Xu J., Wildwater M., Willemsen V., Paponov I., Friml J., Heidstra R., Aida M., Palme K., Scheres B. (2005). The PIN auxin efflux facilitator network controls growth and patterning in Arabidopsis roots. Nature.

[35] Samakovli D., Roka L., Dimopoulou A., Plitsi P.K., Žukauskaitė A., Georgopoulou P., Novák O., Milioni D., Hatzopoulos P. (2021). HSP90 affects root growth in Arabidopsis by regulating the polar distribution of PIN1. New Phytol..

[36] Wang H.Z., Yang K.Z., Zou J.J., Zhu L.L., Xie Z.D., Morita M.T., Tasaka M., Friml J., Grotewold E., Beeckman T. (2015). Transcriptional regulation of PIN genes by FOUR LIPS and MYB88 during Arabidopsis root gravitropism. Nat. Commun..

[37] Rigó G., Ayaydin F., Tietz O., Zsigmond L., Kovács H., Páy A., Salchert K., Darula Z., Medzihradszky K.F., Szabados L. (2013). Inactivation of plasma membrane–localized CDPK-RELATED KINASE5 decelerates PIN2 exocytosis and root gravitropic response in Arabidopsis. Plant Cell.

[38] Cséplő Á., Zsigmond L., Andrási N., Baba A.I., Labhane N.M., Pető A., Kolbert Z., Kovács H.E., Steinbach G., Szabados L. (2021). The AtCRK5 protein kinase is required to maintain the ROS NO balance affecting the PIN2-mediated root gravitropic response in Arabidopsis. Int. J. Mol. Sci..

[39] Kleine-Vehn J., Friml J. (2008). Polar Targeting and endocytic recycling in auxin-dependent plant development. Annu. Rev. Cell Dev. Biol..

[40] Baster P., Robert S., Kleine-Vehn J., Vanneste S., Kania U., Grunewald W., De Rybel B., Beeckman T., Friml J. (2013). SCF(TIR1/AFB)-auxin signalling regulates PIN vacuolar trafficking and auxin fluxes during root gravitropism. EMBO J..

[41] Gibson C.L., Isley J.W., Falbel T.G., Mattox C.T., Lewis D.R., Metcalf K.E., Muday G.K. (2020). A conditional mutation in SCD1 reveals linkage between PIN protein trafficking, auxin transport, gravitropism, and lateral root initiation. Front. Plant Sci..

[42] Kleine-Vehn J., Ding Z., Jones A.R., Tasaka M., Morita M.T., Friml J. (2010). Gravity-induced PIN transcytosis for polarization of auxin fluxes in gravity-sensing root cells. Proc. Natl. Acad. Sci. USA.

[43] Barbosa I.C.R., Schwechheimer C. (2014). Dynamic control of auxin transport-dependent growth by AGCVIII protein kinases. Curr. Opin. Plant Biol..

[44] Barbosa I.C.R., Hammes U.Z., Schwechheimer C. (2018). Activation and polarity control of PINFORMED auxin transporters by phosphorylation. Trends Plant Sci..

[45] Baba A.I., Rigó G., Ayaydin F., Rehman A.U., Andrási N., Zsigmond L., Valkai I., Urbancsok J., Vass I., Pasternak T. (2018). Functional Analysis of the Arabidopsis thaliana CDPK-Related Kinase Family: AtCRK1 Regulates Responses to Continuous Light. Int. J. Mol. Sci..

[46] Baba A.I., Andrási N., Valkai I., Gorcsa T., Koczka L., Darula Z., Medzihradszky K.F., Szabados L., Fehér A., Rigó G. (2019). AtCRK5 protein kinase exhibits a regulatory role in hypocotyl hook development during skotomorphogenesis. Int. J. Mol. Sci..

[47] Baba A.I., Rigó G., Andrási N., Tietz O., Palme K., Szabados L., Cséplő Á., Palocz-Andresen M., Szalay D., Gosztom A., Sípos L., Taligás T. (2019). Striving Towards Abiotic Stresses: Role of the Plant CDPK Superfamily Members. International Climate Protection.

[48] Baba A.I., Valkai I., Labhane N., Koczka L., Andrási N., Klement E., Darula Z., Medzihradszky K., Szabados L., Fehér A. (2019). CRK5 protein kinase contributes to the progression of embryogenesis of Arabidopsis thaliana. Int. J. Mol. Sci..

[49] Grones P., Abas M., Hajný J., Jones A., Waidmann S., Kleine-Vehn J., Friml J. (2018). (2018). PID/WAG-mediated phosphorylation of the Arabidopsis PIN3 auxin transporter mediates polarity switches during gravitropism. Sci. Rep..

[50] Nakamura M., Nishimura T., Morita M.T. (2019). Bridging the gap between amyloplasts and directional auxin transport in plant gravitropism. Curr. Opin. Plant Biol..

[51] Sukumar P., Edwards K.S., Rahman A., Delong A., Muday G.K. (2009). PINOID kinase regulates root gravitropism through modulation of PIN2-dependent basipetal auxin transport in Arabidopsis. Plant Physiol..

[52] Hu X., Neill S.J., Tang Z., Cai W. (2005). Nitric oxide mediates gravitropic bending in soybean roots. Plant Physiol..

[53] Joo J.H., Bae Y.S., Lee J.S. (2001). Role of auxin-induced reactive oxygen species in root gravitropism. Plant Physiol..

[54] París R., Vazquez M.M., Graziano M., Terrile M.C., Miller N.D., Spalding E.P., Otegui M.S., Casalongué C.A. (2018). Distribution of endogenous NO regulates early gravitropic response and PIN2 localization in Arabidopsis roots. Front. Plant Sci..

[55] Zwiewka M., Bielach A., Tamizhselvan P., Madhavan S., Ryad E.E., Tan S., Hrtyan M., Dobrev P., Vankovï R., Friml J. (2019). Root adaptation to H2O2-induced oxidative stress by ARF-GEF BEN1- and cytoskeleton-mediated PIN2 trafficking. Plant Cell Physiol..

[56] Gadalla D.S., Braun M., Böhmer M. (2018). Gravitropism in Higher Plants: Cellular Aspects. Gravitational Biology I. SpringerBriefs in Space Life Sciences.

[57] Hensel W., Behnke H.-D., Esser K., Kubitzki K., Runge M., Ziegler H. (1986). Gravi- and Phototropism of higher plants. Progress in Botany.

[58] Wendt M., Kuo-Huang L.-L., Sievers A. (1987). Gravitropic bending of cress roots without contact between amyloplasts and complexes of endoplasmic reticulum. Planta.

[59] White R.G., Sack F.D. (1990). Actin microfilaments in presumptive statocytes of root caps and coleoptiles. Am. J. Bot..

[60] Baluska F., Hasenstein K.H. (1997). Root cytoskeleton: Its role in perception of and response to gravity. Planta.

[61] Blancaflor E.B., Hasenstein K.H. (1997). The Organization of the actin cytoskeleton in vertical and graviresponding primary roots of maize. Plant Physiol..

[62] Collings D.A., Zsuppan G., Allen N.S., Blancaflor E.B. (2001). Demonstration of prominent actin filaments in the root columella. Planta.

[63] Friedman H., Meir S., Halevy A.H., Philosoph-Hadas S. (2003). Inhibition of the gravitropic bending response of flowering shoots by salicylic acid. Plant Sci..

[64] Livanos P., Galatis B., Apostolakos P. (2014). The interplay between ROS and tubulin cytoskeleton in plants. Plant Signal. Behav..

[65] Blancaflor E.B. (2013). Regulation of plant gravity sensing and signaling by the actin cytoskeleton. Am. J. Bot..

[66] Nakamura M., Toyota M., Tasaka M., Morita M.T. (2011). An Arabidopsis E3 ligase, SHOOT GRAVITROPISM9, modulates the interaction between statoliths and F-actin in gravity sensing. Plant Cell.

[67] Sakai T., Mochizuki S., Haga K., Uehara Y., Suzuki A., Harada A., Wada T., Ishiguro S., Okada K. (2012). The WAVY GROWTH 3 E3 ligase family controls the gravitropic response in Arabidopsis roots: WAV3 family role in root gravitropism. Plant J..

[68] Taniguchi M., Furutani M., Nishimura T., Nakamura M., Fushita T., Iijima K., Baba K., Tanaka H., Toyota M., Tasaka M. (2017). The Arabidopsis LAZY1 family plays a key role in gravity signaling within statocytes and in branch angle control of roots and shoots. Plant Cell.

[69] Furutani M., Morita M.T. (2021). LAZY1-LIKE-mediated gravity signaling pathway in root gravitropic set-point angle control. Plant Physiol..

[70] Mullen J.L., Hangarter R.P. (2003). Genetic analysis of the gravitropic set-point angle in lateral roots of Arabidopsis. Adv. Space Res..

[71] Roychoudhry S., Del Bianco M., Kieffer M., Kepinski S. (2013). Auxin controls gravitropic setpoint angle in higher plant lateral branches. Curr. Biol..

[72] Roychoudhry S., Kepinski S. (2015). Shoot and root branch growth angle control-the wonderfulness of lateralness. Curr. Opin. Plant Biol..

[73] Rosquete M.R., von Wangenheim D., Marhavý P., Barbez E., Stelzer E.H., Benková E., Maizel A., Kleine-Vehn J. (2013). An auxin transport mechanism restricts positive orthogravitropism in lateral roots. Curr. Biol..

[74] Furutani M., Hirano Y., Nishimura T., Nakamura M., Taniguchi M., Suzuki K., Oshida R., Kondo C., Sun S., Kato K. (2020). Polar recruitment of RLD by LAZY1-like protein during gravity signaling in root branch angle control. Nat. Commun..

[75] Harrison B.R., Masson P.H. (2007). ARL2, ARG1 and PIN3 define a gravity signal transduction pathway in root statocytes: ARL2 and ARG1 modulate gravity signal transduction. Plant J..

[76] Baldwin K.L., Strohm A.K., Masson P.H. (2013). Gravity sensing and signal transduction in vascular plant primary roots. Am. J. Bot..

[77] Sedbrook J., Boonsirichai K., Chen R., Hilson P., Pearlman R., Rosen E., Rutherford R., Batiza A., Carroll K., Schulz T. (1998). Molecular genetics of root gravitropism and waving in Arabidopsis thaliana. Gravit Space Biol Bull..

[78] Porterfield D.M., Neichitailo G.S., Mashinski A.L., Musgrave M.E. (2003). Spaceflight hardware for conducting plant growth experiments in space: The early years 1960–2000. Adv. Space Res..

[79] Harvey B., Zakutnyaya O. (2011). Russian Space Probes: Scientific Discoveries and Future Missions.

[80] Vandenbrink J.P., Kiss J.Z. (2016). Space, the Final Frontier: A critical review of recent experiments performed in microgravity. Plant Sci..

[81] Sathasivam M., Hosamani R., Swamy B.K., Kumaran G.S. (2021). Plant responses to real and simulated microgravity. Life Sci. Space Res..

[82] Wolff S., Coelho L., Karoliussen I., Jost A.I. (2014). Effects of the extraterrestrial environment on plants: Recommendations for future space experiments for the MELiSSA higher plant compartment. Life.

[83] Kiss J.Z., Aanes G., Schiefloe M., Coelho L.H.F., Millar K.D.L., Edelmann R.E. (2014). Changes in operational procedures to improve spaceflight experiments in plant biology in the European modular cultivation system. Adv. Space Res..

[84] Braun M., Böhmer M., Häderc D.P., Hemmersbach R., Palme K. (2018). Gravitational Biology I: Gravity Sensing and Graviorientation in Microorganisms and Plants.

[85] Vandenbrink J.P., Kiss J.Z., Herranz R., Medina F.J. (2014). Light and gravity signals synergize in modulating plant development. Front. Plant Sci..

[86] Sachs J. (1882). Über orthotrope und plagiotrope Pflanzenteile. Arb. Bot. Inst. Wurzburg..

[87] Kamal K.Y., Herranz R., van Loon J.J.W.A., Christianen P.C.M., Medina F.J. (2016). Evaluation of simulated microgravity environments induced by diamagnetic levitation of plant cell suspension cultures. Microgravity Sci. Technol..

[88] Hasenstein K.H., van Loon J.J.W.A., Beysens D.A., van Loon J.J.W.A. (2015). Clinostats and other rotating systems—design, function, and limitations. Generation and Applications of Extra-Terrestrial Environments on Earth.

[89] Hemmersbach R., von der Wiesche M., Seibt D. (2006). Ground-based experimental platforms in gravitational biology and human physiology. Signal Transduct..

[90] Hoson T., Kamisaka S., Masuda Y., Yamashita M., Buchen B. (1997). Evaluation of the three-dimensional clinostat as a simulator of weightlessness. Planta.

[91] Kraft T.F.B., van Loon J.J.W.A., Kiss J.Z. (2000). Plastid position in Arabidopsis columella cells is similar in microgravity and on a Random-Positioning Machine. Planta.

[92] Herranz R., Anken R., Boonstra J., Braun M., Christianen P.C.M., de Geest M., Hauslage J., Hilbig R., Hill R.J.A., Lebert M. (2013). Ground-based facilities for simulation of microgravity: Organism-specific recommendations for their use, and recommended terminology. Astrobiology.

[93] Huang B., Li D.G., Huang Y. (2018). Effects of spaceflight and simulated microgravity on microbial growth and secondary metabolism. Military Med Res..

[94] Dedolph R.R., Dipert M.H. (1971). The physical basis of gravity stimulus nullification by clinostat rotation. Plant Physiol..

[95] John S.P., Hasenstein K.H. (2011). Effects of mechanostimulation on gravitropism and signal persistence in Flax roots. Plant Signal. Behav..

[96] Böhmer M., Schleiff E. (2019). Microgravity research in plants A range of platforms and options allow research on plants in zero or low gravity that can yield important insights into plant physiology. EMBO Rep..

[97] Pletser V., Russomano T. (2020). Research in Microgravity in Physical and Life Sciences: An Introduction to Means and Methods. Preparation of Space Experiments.

[98] Limbach C., Hauslage J., Schäfer C., Braun M. (2005). How to activate a plant gravireceptor. Early mechanisms of gravity sensing studied in Characean rhizoids during parabolic flights. Plant Physiol..

[99] Halstead T.W., Dutcher F.R. (1987). Plants in space. Annu. Rev. Plant. Physiol..

[100] Kiss J.Z., Blancaflor E.B. (2015). Conducting plant experiments in space. Plant Gravitropism.

[101] Carillo P., Morrone B., Fusco G.M., De Pascale S., Rouphael Y. (2020). Challenges for a sustainable food production system on board of the International Space Station: A technical review. Agronomy.

[102] Zabel P., Bamsey M., Schubert D., Tajmar M. (2016). Review and analysis of over 40 years of space plant growth systems. Life Sci. Space Res..

[103] Khodadad C.L.M., Hummerick M.E., Spencer L.E., Dixit A.R., Richards J.T., Romeyn M.W., Smith T.M., Wheeler R.M., Massa G.D. (2020). Microbiological and nutritional analysis of lettuce crops grown on the International Space Station. Front. Plant Sci..

[104] Paul A.L., Popp M.P., Gurley W.B., Guy C., Norwood K.L., Ferl R.J. (2005). Arabidopsis gene expression patterns are altered during spaceflight. Adv. Space Res..

[105] Paul A.L., Zupanska A.K., Ostrow D.T., Zhang Y., Sun Y., Li J.-L., Shanker S., Farmerie W.G., Amalfitano C.E., Ferl R.J. (2012). Spaceflight transcriptomes: Unique responses to a novel environment. Astrobiology.

[106] Paul A.L., Zupanska A.K., Schultz E.R., Ferl R.J. (2013). Organ-specific remodeling of the Arabidopsis transcriptome in response to spaceflight. BMC Plant Biol..

[107] Kwon T., Sparks J.A., Nakashima J., Allen S.N., Tang Y., Blancaflor E.B. (2015). Transcriptional response of Arabidopsis seedlings during spaceflight reveals peroxidase and cell wall remodeling genes associated with root hair development. Am. J. Bot..

[108] Kruse C.P.S., Meyers A.D., Basu P., Hutchinson S., Luesse D.R., Wyatt S.E. (2020). Spaceflight induces novel regulatory responses in Arabidopsis seedling as revealed by combined proteomic and transcriptomic analyses. BMC Plant Bio..

[109] Zeng D., Cui J., Yin Y., Xiong Y., Liu M., Guan S., Cheng D., Sun Y., Lu W. (2021). Metabolomics analysis in different development stages on SP0 generation of rice seeds after spaceflight. Front. Plant Sci..

[110] Paul A.L., Levine H.G., McLamb W., Norwood K.L., Reed D., Stutte G.W., William Wells H., Ferl R.J. (2005). Plant molecular biology in the space station era: Utilization of KSC fixation tubes with RNAlater. Acta Astronaut..

[111] Ferl R.J., Zupanska A., Spinale A., Reed D., Manning-Roach S., Guerra G., Cox D.R., Paul A.-L. (2011). The performance of KSC fixation tubes with RNALater for orbital experiments: A case study in ISS operations for molecular biology. Adv. Space Res..

[112] Colla G., Rouphael Y., Cardarelli M., Mazzucato A., Olimpieri I. (2007). Growth, yield and reproduction of Dwarf tomato grown under simulated microgravity conditions. Plant Biosyst. Int. J. Deal. All Asp. Plant Biol..

[113] Zheng H.Q., Han F., Le J. (2015). Higher plants in space: Microgravity perception, response, and adaptation. Microgravity Sci. Technol..

[114] Merkys A.J., Laurinavičius R.S., Kenstavičien P.F., Nečitailo G.S. (1989). formation and growth of callus tissue of Arabidopsis under changed gravity. Adv. Space Res..

[115] Levine H.G., Krikorian A.D. (1992). Shoot growth in aseptically cultivated Daylily and Haplopappus plantlets after a 5-day spaceflight. Physiol Plant.

[116] Salmi M.L., Roux S.J. (2008). Gene expression changes induced by space flight in single-cells of the fern Ceratopteris richardii. Planta.

[117] Hauslage J., Görög M., Krause L., Schüler O., Schäfer M., Witten A., Kesseler L., Böhmer M., Hemmersbach R. (2020). ARABIDOMICS—A new experimental platform for molecular analyses of plants in drop towers, on parabolic flights, and sounding rockets. Rev. Sci. Instrum..

[118] Watanabe C., Fujii N., Yanai K., Hotta T., Kim D.-H., Kamada M., Sasagawa-Saito Y., Nishimura T., Koshiba T., Miyazawa Y. (2012). Gravistimulation changes the accumulation pattern of the CsPIN1 auxin efflux facilitator in the endodermis of the transition zone in cucumber seedlings. Plant Physiol..

[119] Yamazaki C., Fujii N., Miyazawa Y., Kamada M., Kasahara H., Osada I., Shimazu T., Fusejima Y., Higashibata A., Yamazaki T. (2016). The gravity-induced re-localization of auxin efflux carrier CsPIN1 in cucumber seedlings: Spaceflight experiments for immunohistochemical microscopy. npj Microgravity.

[120] Oka M., Kamada M., Inoue R., Miyamoto K., Uheda E., Yamazaki C., Shimazu T., Sano H., Kasahara H., Suzuki T. (2020). Altered localisation of ZmPIN1a proteins in plasma membranes responsible for enhanced-polar auxin transport in etiolated maize seedlings under microgravity conditions in space. Funct. Plant Biol. FPB.

[121] Matía I., González-Camacho F., Herranz R., Kiss J.Z., Gasset G., van Loon J.J.W.A., Marco R., Javier Medina F. (2010). Plant cell proliferation and growth are altered by microgravity conditions in spaceflight. J. Plant Physiol..

[122] Johnson C.M., Subramanian A., Edelmann R.E., Kiss J.Z. (2015). Morphometric analyses of petioles of seedlings grown in a spaceflight experiment. J Plant Res..

[123] Correll M.J., Pyle T.P., Millar K.D.L., Sun Y., Yao J., Edelmann R.E., Kiss J.Z. (2013). Transcriptome analyses of Arabidopsis thaliana seedlings grown in space: Implications for gravity-responsive genes. Planta.

[124] Paul A.L., Sng N.J., Zupanska A.K., Krishnamurthy A., Schultz E.R., Ferl R.J. (2017). Genetic dissection of the Arabidopsis spaceflight transcriptome: Are some responses dispensable for the physiological adaptation of plants to spaceflight?. PLoS ONE.

[125] Choi W.G., Barker R.J., Kim S.-H., Swanson S.J., Gilroy S. (2019). Variation in the transcriptome of different ecotypes of Arabidopsis thaliana reveals signatures of oxidative stress in plant responses to spaceflight. Am. J. Bot..

[126] Paul A.L., Amalfitano C.E., Ferl R.J. (2012). Plant Growth strategies are remodeled by spaceflight. BMC Plant Biol..

[127] Kozeko L.Y., Buy D.D., Pirko Y.V., Blume Y.B., Kordyum E.L. (2018). Clinorotation affects induction of the heat shock response in Arabidopsis thaliana seedlings. Gravit. Space Res..

[128] Aubry-Hivet D., Nziengui H., Rapp K., Oliveira O., Paponov I.A., Li Y., Hauslage J., Vagt N., Braun M., Ditengou F.A. (2014). Analysis of gene expression during parabolic flights reveals distinct early gravity responses in Arabidopsis roots. Plant Biol..

[129] Beisel N.S., Noble J., Barbazuk W.B., Paul A.L., Ferl R.J. (2019). Spaceflight-induced alternative splicing during seedling development in Arabidopsis thaliana. npj Microgravity.

[130] Ferl R.J., Paul A.L. (2016). The effect of spaceflight on the gravity-sensing auxin gradient of roots: GFP reporter gene microscopy on orbit. npj Microgravity.

[131] Babbick M., Dijkstra C., Larkin O.J., Anthony P., Davey M.R., Power J.B., Lowe K.C., Cogoli-Greuterd M., Hamp R. (2007). Expression of Transcription Factors after Short-Term Exposure of Arabidopsis thaliana Cell Cultures to Hypergravity and Simulated Microgravity (2-D/3-D Clinorotation, Magnetic Levitation). Adv. Space Res..

[132] Sheppard J., Land E.S., Toennisson T.A., Doherty C.J., Perera I.Y. (2021). 2021. Uncovering transcriptional responses to fractional gravity in Arabidopsis roots. Life.

[133] Manzano A., Herranz R., den Toom L.A., te Slaa S., Borst G., Visser M., Medina J., van Loon J.J. (2018). Novel, Moon and Mars, partial gravity simulation paradigms and their effects on the balance between cell growth and cell proliferation during early plant development. npj Microgravity.

[134] Kamal K.Y., Herranz R., van Loon J.J., Medina F.J. (2019). Cell cycle acceleration and changes in essential nuclear functions induced by simulated microgravity in a synchronized Arabidopsis cell culture. Plant Cell Environ..

[135] Jiao S., Hilaire E., Paulsen A.Q., Guikema J.A. (2004). Brassica rapa plants adapted to microgravity with reduced photosystem I and its photochemical activity. Physiol. Plant..

[136] Jagtap S.S., Awhad R.B., Santosh B., Vidyasagar P.B. (2011). Effects of Clinorotation on Growth and Chlorophyll Content of Rice Seeds. Microgravity Sci. Technol..

[137] Kitaya Y., Kawai M., Tsuruyama J., Takahashi H., Tani A., Goto E., Saito T., Kiyota M. (2001). The effect of gravity on surface temperature and net photosynthetic rate of plant leaves. Adv. Space Res..

[138] Faraoni P., Sereni E., Gnerucci A., Cialdai F., Monici M., Ranaldi F. (2019). Glyoxylate cycle activity in Pinus pinea seeds during germination in altered gravity conditions. Plant Physiol. Biochem..

[139] Ranaldi F., Vanni P., Giachetti E. (2003). Enzyme catalysis in microgravity: Steady-state kinetic analysis of the isocitrate lyase reaction. Biophys. Chem..

[140] Chandler J.O., Haas F.B., Khan S., Bowden L., Ignatz M., Enfissi E.M., Gawthrop F., Griffiths A., Fraser P.D., Rensing S.A. (2020). Rocket science: The effect of spaceflight on germination physiology, ageing, and transcriptome of Eruca sativa seeds. Life.

[141] Zeng D., Cui J., Yin Y., Zhang M., Shan S., yao Liu M., Cheng D., Lu W., Sun Y. (2020). Proteomic analysis in different development stages on SP0 generation of rice seeds after space flight. Life Sci. Space Res..

[142] Villacampa A., Sora L., Herranz R., Medina F.J., Ciska M. (2021). Analysis of gravi-response and biological effects of vertical and horizontal clinorotation in Arabidopsis thaliana root tip. Plants.

[143] Villacampa A., Fañanás-Pueyo I., Medina F.J., Ciska M. (2022). Root growth direction in simulated microgravity is modulated by a light avoidance mechanism mediated by flavonols. Physiol. Plant..

[144] Aronne G., Muthert L.W.F., Izzo L.G., Romano L.E., Iovane M., Capozzi F., Manzano A., Ciska M., Herranz R., Medina F.J. (2022). A novel device to study altered gravity and light interactions in seedling tropisms. Life Sci. Space Res..

[145] Oluwafemi F.A., Akpu S.U., Akomolafe C.B., Billyok B.J., Okhuelegbe E.O., Doherty K.B., Olubiyi R., Adeleke O., Oluwafemi L., Agboola O.A. (2022). Microgravity-simulation of plant growth and its implications to the Sustainable Development Goals. Int. J. Biomed. Health Sci..

[146] Xu P., Chen H., Hu J., Pang X., Jin J., Cai W. (2022). Pectin methylesterase gene AtPMEPCRA contributes to physiological adaptation to simulated and spaceflight micro-gravity in Arabidopsis. iScience.

[147] Mohammadalikhani S., Ghanati F., Hajebrahimi Z., Sharifi M. (2022). Molecular and biochemical modifications of suspension-cultured tobacco cell walls after exposure to alternative gravity. Plant Physiol. Biochem..

